# The interplay mechanisms between gut microbiota and ferroptosis in inflammatory bowel disease

**DOI:** 10.3389/fimmu.2026.1753617

**Published:** 2026-01-21

**Authors:** Zihan Shi, Shuyun Zhang, Hongru Zhai, Qingmin Wu, Yanyun Li, Huibin Xu, Shanlong Zhang

**Affiliations:** Department of Clinical Laboratory, The Second Affiliated Hospital of Shandong First Medical University, Taian, Shandong, China

**Keywords:** ferroptosis, gut microbiota, iron, iron homeostasis, lipid peroxidation

## Abstract

Inflammatory bowel disease (IBD) is a chronic relapsing disorder driven by complex interactions between genetic susceptibility, immune dysregulation, and environmental factors. Ferroptosis has been identified as a key regulator in the progression of IBD. While much research focuses on endogenous signaling pathways, extrinsic mechanisms—particularly the modulation of IBD through the gut microbiota-induced ferroptosis remain underexplored. Dysregulated ferroptosis, influenced by gut microbiota, exacerbates microbial imbalance, creating a vicious cycle. Notably, the gut microbiota plays a critical role in IBD progression through multidimensional mechanisms, including regulation of metabolites, maintenance of immune homeostasis, and protection of the intestinal barrier. This review examines the microbiota–ferroptosis axis in IBD pathogenesis, aiming to provide insights into potential therapeutic strategies. In particular, we discuss emerging treatments targeting ferroptosis inhibition, iron homeostasis regulation, and microbiota interventions, which hold promise for improving clinical outcomes and promoting pathological recovery in IBD patients.

## Graphical Abstract

**Figure:**
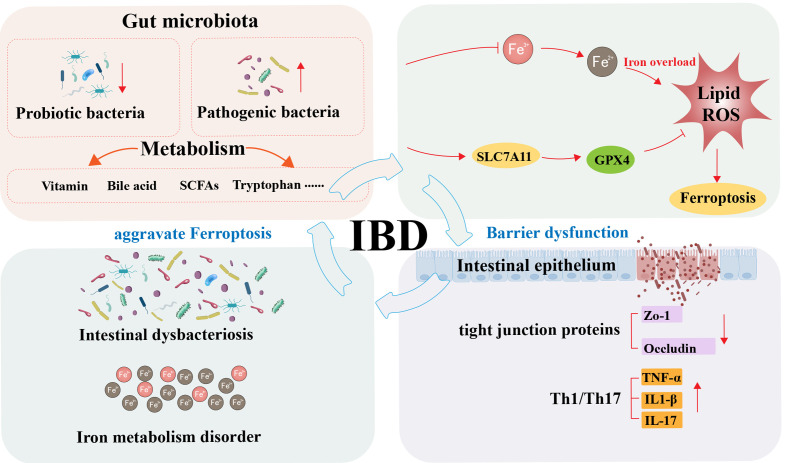
Schematic illustration of the interplay mechanisms between gut microbiota and ferroptosis in inflammatory bowel disease.

## 1 Introduction

Inflammatory bowel disease (IBD) is an immune-mediated disease characterized by chronic, recurrent inflammation of the intestinal tract. The clinical subtypes mainly comprise ulcerative colitis (UC) and Crohn’s disease (CD), and their incidence is particularly significant in adolescents and young adults (1). In the process of IBD, factors such as host genetics, environment, and microbes act on the local intestinal microenvironment, leading to a localized immune response in the intestinal mucosa of genetically susceptible hosts against dysbiosis of the commensal gut microbiota (2). Its typical clinical symptoms include bloody diarrhea, abdominal pain, and progressive weight loss, which seriously impair patients’ quality of life and long-term prognosis (3). Current clinical management strategies for IBD primarily focus on symptom control, with commonly used pharmacological agents including aminosalicylates (5-ASA), corticosteroids, immunomodulators, and biologics (4). Other general measures should be supplemented when necessary, according to the patient’s clinical symptoms (5), and combined surgical intervention should be performed for patients with concurrent intestinal stenosis or perforation (6). However, the existing therapies have significant limitations, often resulting in serious drug side effects and surgical complications. Studies have shown that long-term use of glucocorticoids can induce metabolic syndrome and increase the risk of opportunistic infections. At the same time, immunosuppressants may weaken the efficacy of vaccination and lead to increased susceptibility to viruses (3). Although biologics have improved outcomes in moderate-to-severe IBD, their high cost can impose a substantial financial burden, prompting growing interest in biosimilars with comparable efficacy and safety at lower cost. Moreover, most biologics are administered parenterally, which may increase the risks of infections and hypersensitivity and complicate long-term management (7). What is particularly serious is that with the increasing global prevalence of IBD and the aggravation of drug resistance, the conventional treatment modalities are facing a bottleneck, and it is urgent to explore novel therapeutic strategies.

Ferroptosis, a novel form of programmed cell death, differs from traditional types, including apoptosis, pyroptosis, and autophagic cell death. It involves multiple physiological metabolic processes, including iron metabolism, lipid metabolism, oxidative stress, amino acid metabolism, and biosynthesis. It is primarily manifested by increased intracellular iron levels and enhanced lipid peroxidation, ultimately leading to cell death characterized by mitochondrial shrinkage, loss of cristae, and disruption of membrane integrity (8). Ferroptosis has been implicated in the pathogenesis of a broad spectrum of diseases, including inflammatory diseases (9, 10), cancer (11, 12), neurodegenerative (13, 14), and cardiovascular diseases (15), as well as infectious and systemic illnesses (16). Studies have demonstrated that targeting ferroptosis with pathway-specific inhibitors or activators can help ameliorate disease progression (17). However, excessive ferroptosis exacerbates colitis symptoms, as confirmed in both a dextran sulfate sodium (DSS)-induced murine model and clinical specimens from IBD patients. Administration of ferroptosis-related factor inhibitors has demonstrated therapeutic efficacy in ameliorating IBD (18). The proposed mechanism may involve several pathways, including the inhibition of ferroptosis-related proteins, enhancement of local iron metabolism, and restoration of the intestinal epithelial barrier integrity.

The Gut Microbiota, as a vital component of the intestinal microenvironment, plays a pivotal role in maintaining intestinal homeostasis, modulating host immune responses, participating in metabolic regulation, and promoting nutrient absorption. Breakthroughs in multi-omics technologies have confirmed that gut microbiota and their metabolites play a crucial role in regulating the pathological progression of IBD (19). According to the latest research, IBD is considered to result from abnormal immune responses triggered by genetically susceptible hosts against intestinal symbiotic microorganisms (20). The gut microbiome serves as a metabolic organ, promoting host wellness by executing a variety of biological activities. Alterations in the gut microbiome composition can lead to several pathological conditions, including IBD (21). This theory is of great significance in promoting the transformation of clinical IBD treatment strategies to microbial-targeted therapy. Currently, microecological regulatory therapies, represented by precise probiotic interventions and standardized fecal microbiota transplantation, have demonstrated promising clinical potential in alleviating intestinal inflammation through the restoration of gut microbiota homeostasis (22). However, the precise molecular mechanisms through which the gut microbiota contributes to IBD development are not fully understood. In particular, the regulation of ferroptosis in intestinal epithelial cells by microbial metabolites—such as short-chain fatty acids (SCFAs) and bile acids—through epigenetic modifications and metabolic reprogramming requires further investigation. The association between microbiome dysbiosis (including the bacteriome, virome, and mycobiome) and IBD progression is an area of growing interest. Given the critical role of ferroptosis in intestinal epithelial damage and the amplification of inflammatory responses, targeting the gut microbiota–ferroptosis axis may represent a novel therapeutic direction for IBD in the future.

This review focuses on describing local intestinal iron metabolism, the regulation of iron homeostasis, and host-microbiota interactions based on current research findings, while also elucidating the underlying molecular mechanism of metabolite-modulated ferroptosis plasticity. Additionally, it discusses strategies to reduce the susceptibility of intestinal cells to ferroptosis by targeting these pathways and manipulating them, offering promising avenues for addressing current challenges in IBD treatment.

## 2 Intestinal iron metabolism and regulation

As an essential trace element, iron is integral to numerous biological processes, including oxygen transport, ATP production, immune regulation, DNA synthesis, and repair (23). It is indispensable for proper cellular function. However, paradoxically, iron overload can induce oxidative stress, causing damage to cellular membranes, proteins, and DNA. Which, in turn, may trigger inflammatory responses, apoptosis, and ultimately, tissue destruction. Additionally, oxidative stress impairs immune function and increases susceptibility to infections (24). To maintain functional homeostasis across tissues and cells, the body tightly orchestrates systemic iron balance through the expression and activity of iron carriers, transporters, as well as regulatory and storage proteins (25). A network of hormones, cytokines, and regulatory proteins dynamically sustains this equilibrium.

### 2.1 Intestinal iron absorption and homeostasis

The available iron in the body mainly comes from dietary intake and the phagocytosis of senescent red blood cells by macrophages (26). According to the different forms and absorption mechanisms, dietary iron can be divided into heme iron and non-heme iron. Following dietary intake, the duodenum absorbs non-heme iron primarily as Ferric iron (Fe³^+^) at the brush border membrane of epithelial cells. Duodenal cytochrome B (DcytB), potentially in concert with other reductants, ultimately reduces Fe³^+^ to ferrous iron (Fe²^+^). The reduced Fe^2+^ is then transported into the labile iron pool (LIP) within duodenal cells via divalent metal transporter 1 (DMT1) (27). A portion of Fe^2+^ is stored intracellularly in the form of ferritin, which plays a critical role in the strict regulation of iron absorption (28). Simultaneously, the remaining Fe^2+^ is released from the basal membrane of intestinal epithelial cells into the circulatory system via the ferroportin (FPN). The body mainly absorbs heme iron through endocytosis (29). After entering the small intestinal epithelial cells, free Fe^2+^ is released into the cytoplasm by the heme oxygenase-1 (HO-1) and stored in the active iron pool. When the body requires iron, Fe^2+^ is transported to the portal vein by the ferroportin 1 (FPN1) protein. Plasma ceruloplasmin (CP) and other membrane iron transport auxiliary proteins oxidize these iron ions into Fe³^+^. The resulting iron ions then circulate in the plasma as Fe^3+^, binding with transferrin (Tf) to form iron-transferrin complexes (30). The acidic environment of the endosomes promotes the release of Fe^3+^ from Tf, which is then reduced to Fe^2+^ by prostate six transmembrane epithelial antigen 3 (STEAP3) (31). This process maintains iron in a soluble form, enabling its delivery via transferrin receptors to tissues and cells with functional demand.

The gut exhibits precise regulation of iron homeostasis. These regulatory factors maintain iron concentrations within optimal physiological ranges by controlling iron absorption, storage, and utilization. This process directly meets physiological iron demands without inducing the pathological changes associated with iron overload or deficiency. Research on localized intestinal iron metabolism reveals a connection between oxidative stress within the gut and iron-dependent Fenton reactions. During iron overload, excess Fe^2+^ generates substantial reactive oxygen species (ROS) through the Fenton reaction, inducing localized oxidative stress. This heightens susceptibility to the toxic effects of iron overload, disrupting intestinal mucosal homeostasis and triggering local inflammation and cell death, ultimately precipitating the onset of gut-associated diseases (32).

### 2.2 Iron overload, deficiency, and IBD pathogenesis

In the intestinal microenvironment of IBD, iron overload or functional iron deficiency not only serves as a source of oxidative stress but also acts as a critical factor triggering ferroptosis in intestinal epithelial cells. When intestinal inflammation disrupts the body’s iron balance, abnormal iron metabolism may lead to the formation of excessive activated iron, causing iron deposition and lipid peroxidation. This heightens susceptibility to the toxic effects of iron overload, disrupting intestinal mucosal homeostasis and triggering local inflammation and cell death, ultimately precipitating the onset of gut-associated diseases (8, 32).

Iron overload-related diseases rank among the most prevalent genetic disorders in humans, characterized pathologically by systemic iron accumulation resulting from excessive dietary iron absorption and iron-induced oxidative stress responses (33). Excess iron catalyzes the production of ROS through the Fenton reaction, disrupting colonic mucosal homeostasis, compromising epithelial integrity, and impairing the gut microbiota interaction. These mechanisms exacerbate colonic inflammation and may even promote colorectal cancer development (34). Conversely, while iron deficiency may partially inhibit pathogen growth by limiting bacterial iron uptake, it simultaneously leads to iron-deficiency anemia and compromises intestinal barrier function (35). As the most common form of anemia globally, iron-deficiency anemia (IDA) develops from chronic iron deficiency, persistent blood loss, or impaired iron absorption (36). Notably, the prevalence of concomitant iron deficiency anemia in IBD patients reaches 6–74% (37). Although iron supplementation is the standard treatment for IDA, its use in IBD patients requires caution. Oral iron supplements may disrupt local iron balance due to intestinal free iron accumulation, which increases oxidative stress and ultimately worsens IBD progression (38).

## 3 Ferroptosis and inflammatory bowel disease

The imbalance in ferroptosis regulation plays a key role in the pathogenesis of various diseases. Evidence clearly indicates that ferroptosis plays a pivotal regulatory role in intestinal disorders. Specifically, gut dysfunction is closely associated with ferroptosis, which exhibits dual roles across different cell types and disease contexts. It may function as a positive regulator of intestinal disease while also undertaking negative regulatory functions (39). In IBD, abnormal activation of ferroptosis serves as a critical driver of disease progression. Studies indicate that inhibiting ferroptosis can effectively alleviate IBD-related pathological changes, with protective mechanisms including reduced inflammatory cell infiltration, decreased levels of pro-inflammatory factors, and maintenance of intestinal epithelial barrier integrity. Given these findings, targeted regulation of ferroptosis has emerged as a highly promising novel therapeutic strategy in IBD clinical management.

### 3.1 Ferroptosis

**Figure 1:**
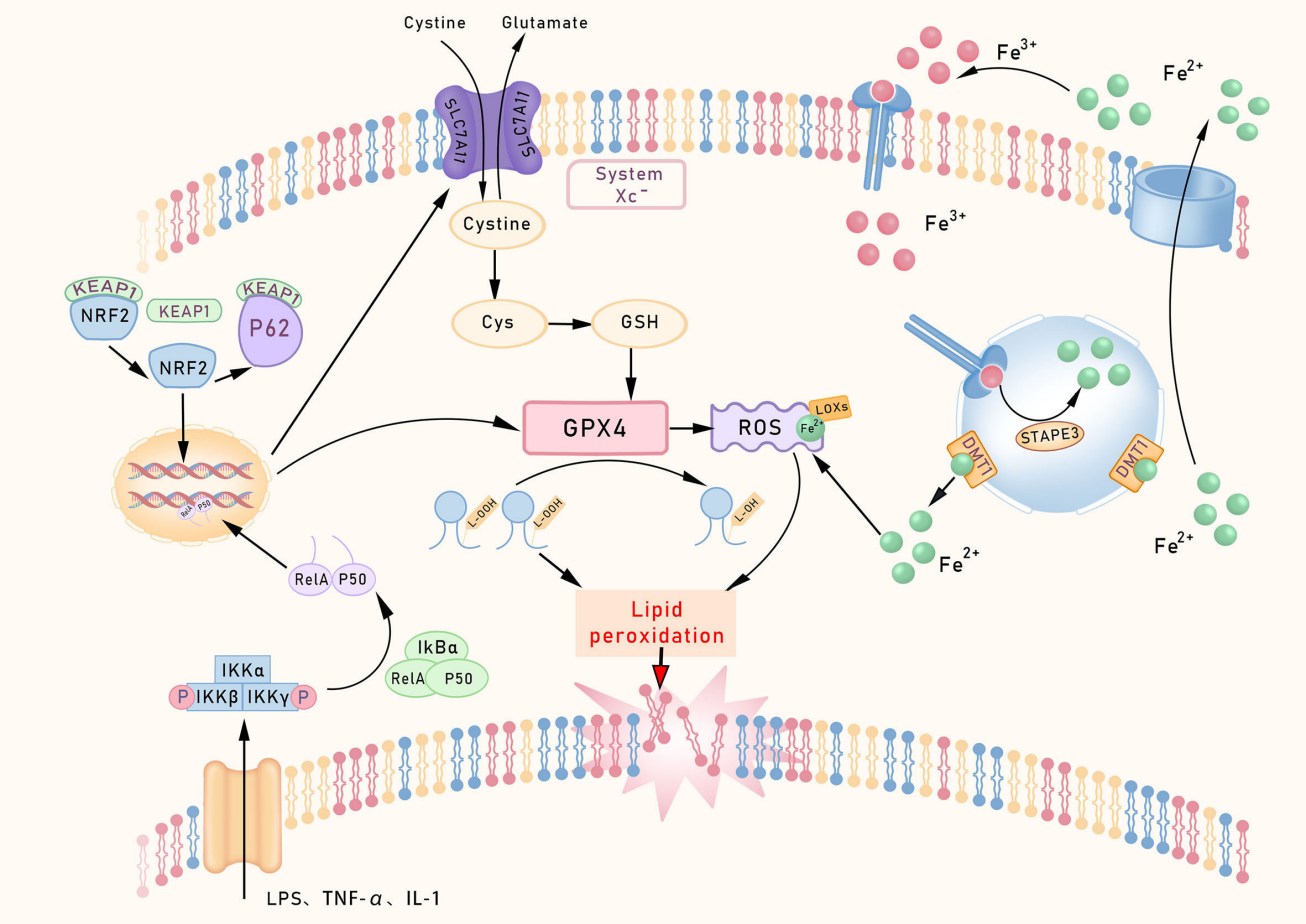
Core regulatory pathways of ferroptosis. This schematic outlines the core ferroptosis regulatory network. The System Xc^–^GPX4 axis constitutes the primary defense, utilizing GSH to neutralize lipid peroxides. Iron metabolism supplies catalytic Fe²^+^ via TFR1/STEAP3 to drive Fenton reactions. Lipid peroxidation is initiated by ACSL4/LPCAT3-mediated incorporation of PUFAs into membranes, rendering them susceptible to LOX-mediated oxidation. Transcriptionally, NF-κB promotes, while NRF2 inhibits ferroptosis. The FSP1-CoQ10 axis provides a parallel defense by generating antioxidant ubiquinol (CoQ10H_2_). TfR1, Transferrin receptor 1; p53 Protein p53.

Ferroptosis is a form of regulated cell death characterized by its high dependence on iron; its core mechanism involves iron ion-catalyzed lipid peroxidation. When excessive iron ions accumulate in cells, the resulting free Fe²^+^triggers Fenton reactions that generate ROS. These ROS then attack phospholipids rich in polyunsaturated fatty acids (PUFAs) on cell membranes, leading to the accumulation of lipid peroxides (LPOs) and compromising membrane integrity (8). Growing evidence suggests that the ferroptosis process is regulated by a complex signaling network involving glutathione (GSH) metabolism, iron metabolism, and the control of oxidative stress (**Figure 1**).

One of the core characteristics of ferroptosis is the collapse of the antioxidant defense system. Glutathione peroxidase 4 (GPX4), a key enzyme in lipid peroxide repair, reduces lipid peroxides by consuming GSH, thereby converting potentially toxic lipid hydroperoxides into non-toxic lipid alcohols (40). When GPX4 activity is lost, or its substrate GSH becomes increasingly depleted, ferroptosis progresses more rapidly. The Conrad team (41) employed a conditional GPX4 knockout mouse model to investigate the role of GPX4 in ferroptosis, revealing that GSH levels and GPX4 activity are key regulators of this process, whose depletion or inactivation in intestinal tissues leads to unchecked lipid peroxidation and epithelial cell vulnerability, a hallmark observed in IBD patients and experimental colitis models (42). Cystine/glutamate antiporter (System Xc-), acting as a System Xc- on the cell membrane, regulates the upstream pathways of ferroptosis by primarily transporting extracellular cystine into the cell for GSH synthesis. Inhibition of System Xc- using sorafenib reduces cystine uptake and indirectly suppresses GPX4 activity by depleting GSH, thereby inducing ferroptosis (43).

In recent years, understanding of ferroptosis-related mechanisms has progressively deepened. In 2019, Sebastian Doll’s team (44) identified ferroptosis suppressor protein 1(FSP1) as a key molecule mediating ferroptosis resistance via a GPX4-independent pathway. Its expression levels correlate strongly with cellular resistance to ferroptosis. Even in cells lacking GPX4 or GSH, the FSP1/CoQ10 pathway still provides ferroptosis protection, forming a ‘dual safeguard’ for cellular defense. Building upon this, the team further elucidated that FSP1’s inhibitory effect on ferroptosis depends on the mediation of ubiquinone, also known as coenzyme Q10 (CoQ10). FSP1 reduces exogenous CoQ10 to its hydroquinone form (CoQ10H2), which subsequently eliminates lipid peroxides to suppress ferroptosis and ultimately alleviate intestinal ischemia-reperfusion (I/R) injury, establishing the FSP1/CoQ10 axis as a defined molecular mechanism against ferroptosis. Furthermore, the study demonstrates that the anti-ferroptotic effect of CoQ10 in intestinal epithelial cells (IECs) is strictly FSP1-dependent, as FSP1 knockout markedly sensitizes IECs to ferroptosis (45). These findings suggest the potential for the combined use of GPX4 and FSP1 activators or inhibitors to regulate ferroptosis in the treatment of intestinal disorders in a synergistic manner.

The gut exhibits heightened susceptibility to oxidative stress due to its unique physiological structure and function (46). In response to such stress, Nuclear factor erythroid 2-related factor 2 (NRF2) acts as a key transcription factor, coordinating multiple intracellular antioxidant defense systems by directly or indirectly regulating the expression of downstream target genes such as GPX4, thereby safeguarding cells from ferroptosis. Under resting conditions, the KEAP1 protein strictly represses the activity of NRF2. Its protein levels are maintained at low levels through the ubiquitin-proteasome pathway, mediated by Kelch-like ECH-associated protein 1 (Keap1) (47). Meanwhile, the core NF-κB signaling complex (typically composed of RelA, IκBα, and p50 subunits) remains inactive in the cytoplasm. When cells encounter oxidative stress or ferroptosis-inducing stimuli, a series of activation events is triggered (48).

The NRF2 protein initiates multiple activation pathways, including nuclear translocation. On one hand, such stimuli can activate the IKK complex (IKKα/IKKβ/IKKγ) through pathways like TNF-R1–TRAF2 or ROS-RIPK1. Activated IKKβ further phosphorylates Ser32/36 sites on IκBα, leading to its ubiquitin-dependent degradation. This process releases the inhibition on the NF-κB (RelA/p50) dimer, exposing the nuclear localization signal (NLS) of the RelA/p50 dimer and driving its nuclear translocation. On the other hand, the key stress response adapter protein p62 competitively binds to Keap1 through its N-terminal Keap1-interacting region (KIR motif), forming a p62-Keap1 complex. This interaction blocks Keap1’s ubiquitin-dependent degradation of NRF2, stabilizing the protein and promoting its nuclear translocation and transcription of target genes, thereby forming a p62-Keap1-NRF2 positive feedback loop. Nuclear translocation of NRF2 upregulates its own expression and that of the p62 gene (also known as SQSTM1). The newly synthesized p62 protein further binds to and inhibits Keap1, thereby amplifying the NRF2 signaling pathway (49). Following nuclear translocation, NRF2 significantly enhances cellular antioxidant defenses by inducing the expression of downstream antioxidant genes, such as GPX4, GCLC, and GCLM (50).

Activation of the NRF2 pathway effectively suppresses intestinal ferroptosis. In clinical applications, numerous natural compounds, acting as endogenous activators of NRF2, demonstrate significant potential in alleviating intestinal disorders by promoting nuclear translocation and the expression of downstream antioxidant and anti-ferroptotic genes (51). NRF2 activation serves as a fundamental cellular protective response. However, its mechanism of action exhibits considerable complexity. Under conditions such as chronic inflammation or specific tissue microenvironments, NRF2 activation may promote ferroptosis by upregulating genes involved in iron metabolism and lipid peroxidation, particularly when antioxidant defenses are overwhelmed. Research suggests that Astragalus polysaccharides can downregulate overactivated NRF2/HO-1 signaling, restoring redox homeostasis and blocking ferroptosis, thereby alleviating the progression of experimental colitis (52). Thus, whether NRF2 acts as a protector or a promoter of ferroptosis in IBD is influenced by factors such as cellular metabolism, microbial metabolites, and tissue-specific conditions.

Uncontrolled lipid peroxidation is a hallmark of ferroptosis, with Acyl-CoA synthetase long-chain family member 4 (ACSL4) playing a central regulatory role in this process. Through whole-genome CRISPR screening, Dixon’s team (53) identified ACSL4 as a gene essential for GPX4-induced ferroptosis. Emerging evidence suggests that inhibition of ACSL4 alleviates epithelial ferroptosis and subsequent inflammation in IBD models (54). Beyond the ACSL4 pathway, mitochondria also actively participate in the regulation of ferroptosis. Additionally, the regulation of ferroptosis involves other critical systems, such as the tetrahydrobiopterin (BH4)-GCH1 system (55) and the dihydrofolate reductase-reduced coenzyme Q (DHFR-CoQH_2_) system (56, 57). FUN14 domain-containing 2 (FUNDC2), a mitochondrial receptor protein, participates in regulating mitochondrial dynamics and metabolic homeostasis.FUNDC2 participates in this regulatory process by interacting with SLC25A11 to modulate mitochondrial GSH levels (58).

The above studies systematically reveal a close connection between ferroptosis and various metabolic pathways, providing a theoretical basis for further understanding its pathological mechanisms in colitis and other related diseases.

### 3.2 Iron dysregulation in modulating ferroptosis susceptibility

Iron overload, as a key inducer of ferroptosis, disrupts cellular iron homeostasis by interfering with critical components of iron metabolism at both transcriptional and post-transcriptional levels, thereby significantly increasing cellular susceptibility to ferroptosis (59). Iron not only constitutes a prerequisite for lipid peroxide accumulation but also directly participates in the execution of ferroptosis (60); in other words, abnormal accumulation of extracellular iron ions under physiological conditions can serve as a natural trigger for ferroptosis.

In IBD, such as UC and CD, accumulation of iron ions has been identified as a key mechanism underlying intestinal epithelial cell damage (61). Inflammatory factors (e.g., TNF-α, IL-6) and ROS associated with IBD impair the capacity of cells to repair lipid peroxidation by inhibiting the activity or expression of GPX4, synergizing with iron overload to significantly lower the threshold for epithelial cells to undergo ferroptosis. Clinical observations suggest that oral iron chelator deferoxamine can promote intestinal epithelial repair and alleviate clinical symptoms in IBD patients (62). Building on these findings, the role of iron ion-mediated ferroptosis in the pathogenesis and treatment of IBD is attracting increasing attention from researchers. The unique regulation of iron homeostasis in the gut serves as a bridge connecting ‘microbial iron regulation’ with ‘local intestinal ferroptosis’.

### 3.3 Ferroptosis and IBD

Acute inflammation constitutes a protective response to infection or tissue injury; yet, excessive or persistent inflammatory reactions may cause tissue damage and even exacerbate disease progression (63). Research by D. J. Cui et al. (64) indicates that the onset of IBD involves the overactivation of ferroptosis. Furthermore, studies have shown that dysregulation of key ferroptosis genes affects disease susceptibility, progression, and severity in the DSS-induced murine colitis model. Clinical trials have demonstrated that ferroptosis inhibitors can significantly alleviate the typical clinical manifestations of IBD, specifically by enhancing intestinal barrier function, promoting weight restoration, optimizing microbial community structure, and reducing the disease activity index (65, 66).

In recent years, research into the regulatory mechanisms of ferroptosis in the progression of intestinal diseases has made significant advancements. Studies have shown that impaired GPX4 function promotes the development of colorectal cancer. Activating GPX4 can significantly reduce ferroptosis in intestinal epithelial cells (IECs) and improve IBD symptoms (66). Relative investigations revealed that the combined use of GPX4 inhibitors and ferroptosis inducers enhances the immunotherapeutic efficacy in colorectal cancer-associated intestinal diseases (67). Additionally, in human and murine IBD models, upregulated ferroptosis-related gene expression and elevated malondialdehyde (MDA) levels confirm the association between ferroptosis and UC (68).

The role of ferroptosis in the pathogenesis of IBD involves multiple pathways, including iron metabolism disorders, intestinal epithelial barrier damage, genetic susceptibility, immune dysregulation, and gut microbiota imbalance. Among upstream regulators of ferroptosis in IBD, hypoxia-inducible factors (HIFs) occupy a unique position by integrating hypoxic stress, iron homeostasis, and inflammatory signaling, thereby setting the threshold at which epithelial ferroptosis is triggered. Intestinal microcirculatory hypoxia is widely recognized as a permissive condition for the onset and progression of UC (69). At the mechanistic level, intracellular iron availability is coordinated by HIF signaling together with iron regulatory proteins (IRPs) (70), linking oxygen sensing to epithelial redox vulnerability.

A central component of this coupling is HIF-2α, an oxygen- and iron-responsive transcription factor that directly regulates key intestinal iron transport genes, including DMT1, Dcytb, and FPN. As a principal transcriptional regulator of intestinal iron transporters, HIF-2α plays a crucial role in maintaining systemic iron balance after birth (71). Importantly, in active intestinal inflammation, HIF-2α may exert context-dependent effects on ferroptosis susceptibility: even under systemic iron deficiency, local inflammatory cues can paradoxically enhance epithelial iron uptake and/or perturb subcellular iron distribution, thereby increasing the labile iron pool and promoting lipid peroxidation. Consistent with isoform specificity, HIF-2α (rather than HIF-1α) enhances iron absorption in mice (72), and human tissue analyses have reported discrete yet overlapping expression patterns of HIF-1α and HIF-2α (73). Although iron-dependent prolyl hydroxylases (PHDs) regulate both isoforms and iron chelation can stabilize HIF-1α and HIF-2α *in vitro* (74), these observations collectively suggest that the gut may implement additional, tissue-specific mechanisms that bias HIF isoform activity and downstream iron handling.

Beyond iron transport, HIF signaling also shapes IBD through cell-type–specific immune programs, which helps reconcile seemingly conflicting findings across models. HIF-2α has been proposed to alleviate inflammation in certain contexts by reducing intracellular iron overload and limiting ferroptosis. The inflammatory microenvironment in active IBD can markedly upregulate HIF-2α in colonic tissues (75). In parallel, HIF-1α exhibits distinct immune regulatory functions depending on lineage: HIF-1α deficiency in myeloid cells alleviates DSS-induced colitis with increased regulatory T cells (Tregs) (76), whereas HIF-1α deficiency in dendritic cells (DCs) exacerbates colitis with reduced Tregs (77). Conditional knockout studies further indicate opposing roles for myeloid HIF-1α and HIF-2α in DSS colitis, where HIF-1α deficiency ameliorates inflammation but HIF-2α deficiency worsens disease (78). Collectively, these data argue that HIF signaling should not be treated as a single “protective” or “pathogenic” pathway; rather, isoform-, cell type-, and stage-dependent wiring likely determines whether HIF shifts the system toward or away from a ferroptosis-permissive state.

Finally, ferroptosis itself can reinforce HIF activation and disease progression. Ferroptotic injury promotes the release of pro-inflammatory mediators and amplifies oxidative stress (79), which can aggravate microcirculatory dysfunction and hypoxia, thereby reactivating HIF programs and further perturbing iron handling. This establishes a self-reinforcing loop, highlighting why precision strategies targeting iron metabolism and HIF–iron coupling may require careful stratification by tissue niche, inflammatory stage, and cellular compartment in colitis.

Downstream of HIF–iron remodeling, ferroptosis in intestinal epithelial cells (IECs) constitutes a key mechanism that converts upstream threshold shifts into epithelial barrier failure and inflammatory amplification in IBD. At the molecular level, this process is shaped by both genetic programs and disease-associated perturbations in ferroptosis checkpoints. Genetic regulation plays a pivotal role in ferroptosis-mediated IBD pathogenesis. Both clinical IBD patients and the DSS-induced murine colitis model exhibit significant alterations in ferroptosis-associated gene expression profiles (80). Mechanistic studies reveal that inflamed intestinal tissues in IBD patients and corresponding animal models display characteristic ferroptosis features, including GSH depletion, suppression of GPX4 activity, and abnormal iron deposition. Ferroptosis is implicated in IBD, particularly in the death of intestinal epithelial cells (68). Through bioinformatics analysis of UC-related genes, we identified that acyl-CoA synthetase family member 2 (ACSF2) exhibited significantly downregulated expression in DSS-induced colitis models, Salmonella typhi colitis models in mice, and various lipopolysaccharide (LPS)-induced colitis models. Notably, the application of the ferroptosis inhibitor Ferrostatin-1 reversed this phenotype (81). These findings suggest that ACSF2 may alleviate inflammatory responses in IBD and delay the progression of experimental colitis by inhibiting ferroptosis pathways.

Given the critical role of ferroptosis in the pathogenesis of colitis, targeting ferroptosis signaling pathways may become a potential therapeutic strategy for IBD. Currently, certain drugs that alleviate oxidative stress and inflammation by eliminating ROS, such as thioproline and N-acetylcysteine, have been applied in IBD clinical treatments (82), providing valuable references for developing novel ferroptosis inhibitors to improve intestinal diseases.

## 4 The regulatory role of gut microbiota dysbiosis in the pathogenesis of IBD

Clinical data indicate a significant positive correlation between gut microbiota dysbiosis and the onset and progression of IBD, manifested specifically as reduced α-diversity, depletion of commensal probiotic bacteria, and abnormal proliferation of pro-inflammatory pathogens (83). Notably, clinical antibiotic use frequently exacerbates colitis symptoms (84), further underscoring the critical role of microbial homeostasis in maintaining intestinal health. Compared to healthy individuals, IBD patients exhibit universally reduced levels of the microbially derived aryl hydrocarbon receptor (AhR), which may also exert effects by inhibiting the NF-κB/p65 signaling pathway (85). AhR agonist supplementation can significantly enhance intestinal barrier integrity and alleviate IBD-related symptoms (86). Within the pathological state of IBD, gut dysbiosis exacerbates intestinal inflammatory responses through multiple mechanisms. This section will explore its potential role in IBD pathogenesis from the perspective of microbe-host interactions, elucidating the critical function of gut dysbiosis in the disease process.

### 4.1 Immune imbalance

Immune imbalance is the key amplifier that converts dysbiosis and epithelial stress into sustained inflammatory signaling, which subsequently perturbs iron regulation and favors ferroptosis-prone states. Following the excessive proliferation of pathogenic bacteria, the cytotoxins they secrete can directly damage intestinal epithelial cells (87). Additionally, they may activate pattern recognition receptors (PRRs), such as Toll-like receptors (TLRs), thereby promoting the release of pro-inflammatory factors (88). Simultaneously, microbial dysbiosis disrupts intestinal immune tolerance, leading to enhanced Th1/Th17 immune responses while impairing the function of Tregs (89). Research by Read et al. (90) further demonstrates that immune cell dysfunction can lead to intestinal immune regulation disorders. Certain probiotics, however, can restore intestinal immune homeostasis by inducing IgG production and modulating T-cell-mediated immune responses, thereby alleviating colitis (91). For instance, Akkermansia muciniphila in the gut can induce immune homeostasis in mice, promote IgG production, and initiate antigen-specific T-cell responses, thereby improving symptoms of DSS-induced colitis (92).

### 4.2 Genetic susceptibility and microbiota interaction

The impact of genetic polymorphism on IBD is primarily mediated by gut microbiota, highlighting the significance of host genetic variations in disease progression. Genetic factors can modulate the pathogenicity of specific bacteria, with certain pathogens being particularly capable of triggering chronic inflammation under specific genetic conditions (93). Emerging research has also highlighted the critical role of epigenetic and post-transcriptional regulation, particularly through microRNAs (miRNAs), which serve as key modulators of immune responses and microbial interactions in IBD, influencing not only immune modulation but also iron metabolism, oxidative stress, and epithelial integrity, all of which are critical to ferroptosis in IBD (94, 95). To date, genetic research has identified over 100 genetic loci associated with IBD susceptibility, many of which regulate the host’s immune response to bacteria, such as NOD2, TLR5, and IL-10. Taking the NOD2 and CYBB genes as examples, both are susceptibility genes for IBD (96, 97). Mutations in the NOD2 gene are particularly crucial in regulating the pathogenesis of CD’s disease (98). Genetic defects in the innate immune system (such as loss of NOD2 function) lead to dysregulation of the host’s immune response to the gut microbiota, constituting a key driver of chronic inflammation (99).

As a pattern recognition receptor (PRR), NOD2 recognizes bacterial peptidoglycan and regulates the expression of antimicrobial peptides by activating the NF-κB signaling pathway, thereby promoting their production. Notably, NOD2 gene mutations are closely associated with structural alterations in the gut microbiota, further demonstrating that IBD development depends on the interaction between the genetic background and microbial communities (100). In individuals with NOD2 mutations, the gut microbiota exhibits compositional and diversity dysregulation, characterized by a decreased abundance of certain beneficial bacteria and an excessive proliferation of harmful bacteria, which may exacerbate intestinal inflammatory responses. Additionally, under specific pathogen-free (SPF) conditions, H. hepaticus can induce chronic colitis in IL-10−/− mice, whereas no corresponding pathological changes were observed in wild-type mice (101).

### 4.3 Microbial translocation and disruption of the intestinal barrier

In the process by which pathogenic symbionts (pathobionts) induce IBD, the stability of the gut microbiota is crucial. Under healthy conditions, commensal bacteria and pathogens within the gut maintain an appropriate dynamic equilibrium. The microbiota regulates the expression of tight junction proteins (such as ZO-1 and occludin) in intestinal epithelial cells by releasing specific metabolites and signaling molecules, thereby safeguarding the structural and functional integrity of the intestinal barrier (86). In IBD patients, significant dysbiosis frequently occurs. This imbalance leads to downregulation of tight junction protein expression, increased intestinal mucosal permeability, and the initiation and exacerbation of inflammatory cascades. For instance, studies reveal an expansion of mucus-degrading bacteria (such as Akkermansia) in the gut of IBD patients. The mucin-degrading enzymes secreted by these bacteria disrupt the mucus layer’s structure, leading to heightened intestinal mucosal permeability (102). Concurrently, the compromised intestinal barrier facilitates the translocation of microbial components, including LPS and flagellar proteins, along with secondary bile acids, into the systemic circulation, thereby triggering systemic inflammatory responses (103). Studies have also revealed that astragaloside IV, derived from traditional Chinese medicine, can enhance the abundance of Akkermansia muciniphila, repair intestinal mucosal barriers, improve the gut immune environment, and reduce LPS blood entry, thereby suppressing systemic inflammatory responses (104).

Studies demonstrate that when TLR5^–/–^ germ-free mice are infected with adherent-invasive Escherichia coli (AIEC) and subsequently exposed to a specific SPF microbial environment, they develop colitis symptoms (105). In contrast, mice inoculated with the altered Schaedler flora (ASF), a pathogen-free microbial community, exhibit no significant intestinal inflammation after AIEC infection (106). This suggests that AIEC induces chronic intestinal inflammation in susceptible hosts by promoting microbial dysbiosis. Specifically, this pathogenic process depends on a complex microbiota defined by specific species, functions, and interactions. Clinical studies indicate that AIEC exhibits synergistic effects during pathogen infection; its colonization exacerbates clinical outcomes in Salmonella typhimurium-induced infectious gastroenteritis, suggesting pathogenic bacteria may modulate acute infection progression (107).

The gut microbiota of IBD patients exhibits a trend towards reduced diversity, manifested explicitly by a decrease in the abundance of commensal Clostridia and a significant enrichment of pathogenic bacteria. These pathogenic bacteria can colonize the gut by exploiting epithelial damage and activate TH1/TH17 immune responses through virulence factors, such as the type III secretion system (108). Meanwhile, fungal species such as Candida and Malassezia secrete candidalysin, which activates IL-1β, thereby exacerbating inflammatory responses (109). Research by G. Pontarollo’s team (102) has revealed a novel mechanism by which gut microbiota modulate intestinal barrier function: symbiotic bacteria activate TLR-2 signaling in the innate immune receptors of intestinal epithelium, thereby downregulating neuropilin-1 (NRP1) and the Hedgehog signaling pathway it regulates, thus impairing intestinal barrier function.

The data mentioned above indicate that the pathogenic potential of specific microorganisms in IBD is subject to multifactorial regulation, encompassing genetic background, environmental factors, and the composition of the gut microbiota. Diet, as another critical determinant, can rapidly modulate the structure and function of the microbiota, thereby exerting specific effects on the pathogenesis of IBD. Research indicates that a high-fat diet can promote the proliferation of pro-inflammatory Enterobacteriaceae by altering bile acid metabolism, thereby inducing pathogenic translocation (110). In IBD mouse models, high-fat and high-sugar feeding lead to dysbiosis, manifested as excessive proliferation of Escherichia coli, accompanied by destruction of the mucosal layer structure and increased intestinal permeability (111).

Interestingly, the gut virome, consisting of a variety of viruses, also contributes to microbial dysbiosis and immune modulation in IBD. Studies suggest that viral communities can interact with gut bacteria, potentially exacerbating inflammation or influencing immune cell behavior (112). Although most studies have concentrated on bacterial dysbiosis, the virome is emerging as an essential player in the pathogenesis of IBD. Exploring the interactions between bacterial and viral communities in the gut may uncover novel therapeutic targets for modulating the immune system and restoring intestinal homeostasis (113).

## 5 The interaction mechanism between gut microbiota and ferroptosis in IBD

The gut microbiota regulates ferroptosis-related signaling pathways through multiple mechanisms, including (i) remodeling luminal iron availability and epithelial iron handling, (ii) rewiring host antioxidant capacity and lipid peroxidation tone via microbial metabolites, and (iii) modulating mucosal immune programs that determine the ferroptosis threshold of distinct cell types. These effects arise from both “direct microbe–host interactions.” Dysbiosis markedly diminishes the physiological tolerance of intestinal tissues to ferroptosis, thereby compromising the intestinal barrier function and exacerbating colitis (114). Conversely, ferroptosis-driven epithelial injury and iron perturbations can reshape the microbial ecosystem, forming a self-amplifying inflammatory loop.

### 5.1 Ferroptosis regulation based on microbiota-host interaction

Iron competition in the gut is a core ecological force that links microbial fitness to host ferroptosis susceptibility. Using high-throughput screening of microbial metabolites, Das et al. (115) reported that microbiota-derived metabolites suppress HIF-2α, a master regulator of intestinal iron absorption, and increase ferritin levels, thereby limiting host iron uptake and potentially reducing the labile iron pool that fuels lipid peroxidation. Related research has demonstrated that Candida albicans accelerates atherosclerosis by activating intestinal HIF-2α signaling (116). Another piece of evidence that intestinal oxygen dynamics plays a key role in ferroptosis-mediated microbe-host interactions is that the oxygen gradient established in the gut under physiological conditions is crucial for maintaining microbial ecological balance. When elevated host oxygenation disrupts the intestinal radial oxygen gradient, it alters the microbial composition, promoting the enrichment of oxygen-tolerant bacteria while significantly suppressing strict anaerobes, such as Anaerostipes, which produce SCFAs (117). Because SCFAs and other anaerobe-associated metabolites contribute to epithelial redox homeostasis, oxygen-driven community shifts may lower antioxidant buffering and thereby increase ferroptotic vulnerability during inflammation (118).

### 5.2 Specific regulation of the ferroptosis pathway by intestinal metabolites

Ferroptosis is driven by an iron metabolism imbalance and antioxidant system collapse, with immune cells exhibiting high heterogeneity in the response process. The gut microbiota influences ferroptosis by modulating the intestinal antioxidant system, serving as a key factor in the pathogenesis and progression of ferroptosis in IBD. The differential susceptibility of various immune cells to ferroptosis determines their roles in iron homeostasis and antioxidant defense. For instance, Dendritic cells (DCs) play a critical role in antitumor immunity by activating T cells, yet ferroptosis inhibits DC maturation and impairs their antitumor function (119). RSL3-induced ferroptosis leads to the loss of DCs’ ability to secrete pro-inflammatory cytokines and further suppresses T cell activation. Proliferator-Activated Receptor Gamma, PPARγ (PPARG/PPARγ) plays a central role in this process, and knockout of PPARG restores DC function (120). Macrophages and intestinal epithelial cells play critical roles in iron metabolism, which can mediate ROS generation (121). While the function of T cells depends on the iron environment. Compared to effector T cells, regulatory T cells (Tregs) exhibit a greater preference for lipid metabolism and demonstrate resistance to ferroptosis, whereas effector T cells rely on glutamine metabolism, rendering them more susceptible to ferroptosis (122). By knocking out ACSL4 in T cells, D. J. Collins et al. found that reduced levels of PUFA-PLs could promote resistance to ferroptosis, while decreased PUFA-PLs in neutrophils could enhance ferroptosis susceptibility, indicating that PUFA-PLs are the primary factors determining ferroptosis sensitivity in lymphocytes and myeloid cells (123).

At the antioxidant level, commensals can reinforce host redox buffering. For example, Bifidobacteria metabolize and produce antioxidant substances such as vitamin K and GSH precursors, which can reduce cellular sensitivity to ferroptosis by regulating lipid metabolism (124). When pathogenic bacteria invade, they effectively mitigate lipid peroxidation-induced PUFA oxidative damage caused by LPS (125). Another metabolic product of intestinal bacteria, urolithins (UA), is produced through the conversion of dietary ellagitannins and ellagic acid by specific gut microbiota. It can activate the Keap1–NRF2/HO-1 axis to suppress lipid peroxidation and ferroptosis (126), and can also enhance mitophagy and dampen excessive inflammation (127), processes that may converge on ferroptosis control. Microbial signals can also affect epithelial stress-response pathways. Intestinal-origin Lactobacillus rhamnosus GG (LGG) activates the intestinal epithelial AKT-STAT signaling pathway, helping to restore a balanced gut microbiota, promote intestinal epithelial cell proliferation, and repair damage (128). In addition, capsaicin (CAP), another gut microbiota metabolite, alleviates ventilator-induced lung injury by activating Sirtuin 3 (SIRT3) to inhibit ferroptosis and maintain mitochondrial redox homeostasis (129).

In summary, Current studies suggest that certain monomeric components derived from traditional Chinese herbal medicines may exert protective effects through mechanisms such as modulating gut microbiota, improving the microenvironment, alleviating oxidative stress, and inhibiting ferroptosis. Paeoniflorin (PA), a monomeric component of traditional Chinese medicine, improves impaired glucose tolerance and myocardial injury symptoms. It exhibits potent effects against ferroptosis and modifies the composition and structure of the gut microbiota, offering protective benefits in diabetic cardiomyopathy mice (130). Zhang et al. (131) demonstrated that supplementing with Lachnospiraceae bacterium strains significantly alleviated neutrophil infiltration and oxidative stress in ethanol-exposed mouse livers, exhibiting remarkable hepatoprotective effects. The differential metabolite N-acetylglutamate (NAG) activates the KEAP1-NRF2 pathway and concurrently inhibits ferroptosis, thereby mediating a protective effect. However, these findings are primarily based on models such as diabetic cardiomyopathy or liver injury. Translating them into effective strategies for the prevention or treatment of IBD remains a promising scientific hypothesis that urgently requires direct validation of efficacy and specific mechanisms in IBD animal models and clinical studies.

### 5.3 Microbial-ferroptosis interaction as a therapeutic target of IBD

Collectively, accumulating studies suggest that the “microbiota–metabolite–ferroptosis axis” contributes to IBD pathogenesis (**Table 1**) (132). Below, we highlight representative microbiota-dependent metabolites that modulate ferroptosis-relevant nodes (iron handling, lipid peroxidation, antioxidant systems) and discuss their translational potential.

**Table 1:** Overview of metabolites and their effect on ferroptosis and IBD progression.

Metabolites and target(s)	Specific types	Related intestinal microbiota	Mechanisms of regulating microbes in ferroptosis	Role of metabolites in IBD
Vitamin [Target(s): GPX4/GSH, NRF2/HO-1, Lipid peroxidation modulators)]	VB12	Bifidobacterium, Lactobacillus (136)	Increase lipogenesis and lipid peroxidation (200).	Maintaining homeostasis of intestinal epithelial cells leads to exacerbation of inflammation (201)
	VB2(Riboflavin)		Mitigate ROS levels and SLC3A2 protein levels (137)	Reduce intestinal epithelial oxidative damage by the antioxidant effect (202)
	Adenine		As a cofactor to combat ferroptosis (203)	Affects IBD progression (204)
	VB5(Pantothenic Acid)		Inhibited inflammatory response and ferroptosis through the SIRT1/NRF2 signaling pathway (205)	Restrain Th17 cell differentiation as well as related autoimmune diseases (206)
	VB6(Pyridoxine)		Upregulate CBS, GSH and GPX4 (207)	Regulates the tryptophan metabolic pathway and affects AhR receptor activation (208)
	VC	Escherichia coli, Lactobacillus (209, 210)	Regulate ferroptosis through the amino acid and carbohydrate metabolic pathways (211)	Clear free radicals, reduce oxidative stress, and relieve intestinal inflammation (82, 212)
	VD	Bifidobacterium longum, Coprococcus, Actinobacteria (213)	Activate NRF2/HO-1 signaling pathway (214)	Regulate immune response and intestinal barrier function (215, 216)
	VE	Cetobacterium (217)	Antioxidant, reduce oxidative stress (212)	Maintain intestinal barrier function (218)
	VK	Clostridium and lactobacillus (219)	Reduce the level of ROS by modulating the expression of antioxidant enzymes (220)	Maintain intestinal coagulation and barrier function through γ-carboxylation (221)
	VA	Lactobacillus (222)	Antioxidant (223)	Enhance intestinal epithelial barrier function (224)
Bile acid [target(s): FXR/GPX4 axis, NRF2 activation (225)]	Primary bile acids include chenodeoxycholic acid, while secondary bile acids include ursodeoxycholic acid and lithocholic acid.	Clostridium scindens and Clostridium sporogenes (226)	Inhibit the FXR receptor to block ACSL4-mediated lipid peroxidation (227)	The deficiency leads to increased proliferation of pathogenic bacteria, thereby exacerbating IBD (228).
SCFAs [target(s): Histone deacetylase (HDAC) inhibition, NF−κB (229, 230)]	Acetate	Paenibacillus polymyxa (231)	Activate the hepatic AMPK/SIRT1/PGC-1α axis to alleviate ferroptosis (232)	Promote Tregs differentiation and alleviate immune hyperactivation (233)
	Propionate	Lactobacillaceae, Ruminococcaceae, and Lachnospiraceae (234)	Cause the imbalance of ROS (235)	Contribute to IBD (236)
	Butyrate		Ameliorate ferroptosis in experimental colitis through NRF2/GPX4 signaling (237)	Maintain the integrity of the intestinal epithelial barrier and reduce inflammatory response (238)
Tryptophan metabolites [target(s): AhR/NRF2 axis, Indoleamine 2,3-dioxygenase (IDO)-kynurenine signaling, antioxidant gene modulation]	IDA	Peptostreptococcus anaerobius (135)	Activate the AhR/NRF2 pathway to inhibit ROS accumulation and ferroptosis (239)	Promote the growth of probiotics and inhibit the growth of pathogens (240)

#### 5.3.1 Vitamin

Microbiota-related vitamins contribute to barrier integrity by regulating tight junctions, epithelial permeability, and epigenetic programs (133, 134). Microbial synthesis or microbial-shaping of vitamin availability (e.g., B vitamins, vitamin K) may influence host redox and immune programs (135, 136). For instance, VB_2_ (riboflavin) alleviates fluoride-induced ferroptosis by regulating the SLC7A11 system and iron metabolism via an IL-17A-independent pathway (137). Vitamin D deficiency is associated with dysbiosis and worsened colitis, while supplementation can improve microbial composition and barrier-related pathways (138). Mechanistically, VD maintains colonic barrier integrity by regulating the abundance of Akkermansia and muciniphila (139). Additionally, VD-related signaling pathways, such as the NF-κB pathway, regulate antimicrobial peptide expression and immune tolerance, thereby optimizing gut microbiota composition (140). Clinical trials further support these conclusions. A cohort study by M. Marangos’ team (141) revealed a positive correlation between serum VD levels and Faecalibacterium abundance in IBD patients. Patients with lower VD levels exhibited more pronounced microbial dysbiosis. Daily VD supplementation significantly increased butyrate-producing bacteria in the intestines of IBD patients, while reducing the abundance of pathogenic bacteria (140).

Vitamin C can modulate microbiota–immune interactions, promote Treg differentiation via epigenetic mechanisms, reduce oxidative stress, and suppress pathogen colonization (142–144).

#### 5.3.2 α-Tocopherol

α-Tocopherol, as a natural phenolic compound, is the most abundant and potent isomeric form of vitamin E in the human body. Compounds within the vitamin E family directly inhibit ferroptosis by competing with the lipoxygenase (LOX) PUFA substrate site. As an effective antioxidant, α-tocopherol can specifically block lipid peroxidation chain reactions and inhibit LOX activity (145). Research confirms that α-tocopherol synergizes with GPX4 through an electron donor-mediated chain-breaking mechanism to jointly maintain cellular membrane lipid redox homeostasis, thereby effectively inhibiting ferroptosis (146).

α-Tocopherol exhibits significant anti-inflammatory activity, with reports indicating that it ameliorates symptoms in experimental IBD models by protecting intestinal barrier function, modulating the gut microbiota, accelerating intestinal tissue healing, and regulating the immune system (147). Furthermore, iron overload significantly reduces α-tocopherol concentrations in mice. Notably, dietary supplementation with vitamin E alleviates the ferroptosis phenotype induced by GPX4 deficiency (148). Mice with GPX4 gene defects rapidly succumb under vitamin E-deficient conditions, whereas reintroduction of vitamin E-supplemented diets for four weeks reverses this phenotype (149). These observations support the concept that nutritional antioxidant status can buffer ferroptotic stress in the gut (150). Moreover, γ-tocopherol, the predominant vitamin E isomer in the American diet, has shown promising therapeutic potential in IBD treatment due to its unique anti-inflammatory properties (151).

On the other hand, arachidonic acid (AA) metabolism is considered a potential target for regulation in CRC. Studies have shown that low doses of AA can promote ferroptosis and enhance the anti-tumor immune effects induced by immune checkpoint blockade (ICB) therapy (55). LOX can oxidize AA at different carbon positions, participating in the regulation of cellular redox homeostasis and thereby influencing ferroptosis. Thus, LOX inhibitors, such as vitamin E family members (tocopherol and tocotrienol), can effectively prevent ferroptosis (145).

#### 5.3.3 Bile acids

High-fat diet (HFD) reshapes microbiota-dependent bile acid metabolism and promotes the accumulation of secondary bile acids such as deoxycholic acid (DCA) (152). DCA can activate HIF-2α signaling, increase DMT1 expression, elevate epithelial Fe²^+^ accumulation, and trigger ferroptosis; ferrostatin-1 reverses these effects (153). Accordingly, disrupted interactions between the gut microbiota and bile acids impair intestinal barrier function and activate inflammation-related signaling pathways, exacerbating the progression of IBD.

Interestingly, the metabolic dysregulation induced by an HFD manifests multifaceted pathogenic effects. In IBD mouse models fed a high-fat, high-sugar diet, prolonged HFD intake not only induces gut microbiota dysbiosis but also compromises the integrity of the intestinal mucosal barrier, increasing intestinal permeability. This alteration further promotes the adhesion and colonization of invasive AIEC, thereby exacerbating intestinal inflammatory responses (154). Such dysregulated microbiota-host interactions may disrupt immune homeostasis by affecting iron metabolism and subsequently reshape the body’s immune tolerance state by driving the differentiation of Tregs.

In the DCA-induced intestinal inflammation process, 16S rRNA gene sequencing results showed significantly reduced intestinal microbial diversity, specifically manifested as increased proportions of Bacteroidetes and decreased proportions of Firmicutes, indicating that DCA-induced microbial imbalance may be a key factor in the development of intestinal inflammation (155). Additionally, HFD can stimulate hepatic bile acid secretion, promoting increased DCA synthesis and excretion. When DCA accumulates excessively in the intestine, it may cause damage to the mucosal barrier, abnormal immune activation, and microbial dysbiosis, ultimately inducing or exacerbating colitis progression (156). Furthermore, Clostridium hiranonis can alleviate DSS-induced colitis by promoting the production of secondary bile acids (157).

#### 5.3.4 SCFAs

SCFAs are reduced in IBD and are tightly linked to barrier integrity and anti-inflammatory programs (158). As key metabolic products of gut symbiotic microorganisms (such as Bacteroides and Lactobacillus), SCFAs not only help maintain the integrity of the intestinal mucosal barrier but also effectively suppress intestinal inflammation. Furthermore, the microbiota optimizes dietary iron bioavailability by producing essential amino acids and SCFAs, thereby lowering the pH within the intestinal lumen. For instance, the ingestion of non-digestible carbohydrates, such as fructooligosaccharides, promotes SCFA production, which lowers intestinal pH and reduces iron to soluble Fe²^+^ forms. This may further alter ligand composition (159, 160), potentially constituting a key mechanism for high-fat diet-mediated iron overload.

Butyrate, serving as the primary energy source for intestinal epithelial cells, not only maintains colonic barrier function but also alleviates inflammatory responses by inhibiting the NF-κB signaling pathway (161, 162). Gamma-aminobutyric acid (GABA), a derivative of butyric acid derived from gut microbiota, alleviates hepatic ischemia-reperfusion injury by inhibiting ferroptosis. However, antibiotic treatment eliminates the beneficial effects of GABA by depleting gut bacteria (163). Butyrate also promotes Treg homeostasis and may intersect with iron-related pathways relevant to mucosal tolerance (164).

In addition to butyrate, another short-chain fatty acid, valerate, may also enhance iron absorption through multiple mechanisms. For instance, valerate can lower intestinal pH, thereby promoting the reduction of Fe³^+^ to the more bioavailable Fe²^+^ form while simultaneously increasing iron solubility; Secondly, it may activate short-chain fatty acid receptors (SCFA receptors) and inhibit histone deacetylase (HDAC) activity, thereby lifting transcriptional repression on key iron absorption genes (such as Dcytb and Ferroportin) and consequently upregulating intestinal iron uptake.

Furthermore, propionate in SCFAs demonstrates protective effects on the cardiovascular system. Through mechanisms of gut microbiota remodeling, it significantly inhibits vascular calcification (165). Remarkably, low dietary fiber intake reduces beneficial bacteria capable of fermenting dietary fiber to produce SCFAs. SCFAs possess anti-inflammatory properties, promoting the differentiation of Tregs and enhancing intestinal barrier function. Consequently, a low-fiber diet diminishes the gut’s anti-inflammatory capacity, increasing the risk of IBD. Overall, the high intake of fat, sugar, and carbohydrates characteristic of Western dietary patterns correlates positively with IBD risk (166).

#### 5.3.5 Selenium

Selenium is an essential trace element for the human body, whose core biological function lies in its role as a constituent of selenocysteine (Sec). Selenocysteine serves as a key component of the active centers in numerous selenoproteins. Within the selenoprotein family, GPX4 has garnered significant attention due to its pivotal role in catalyzing the reduction of hydrogen peroxide and lipid peroxides, thereby maintaining cellular redox homeostasis (167). Notably, the enzymatic activity of GPX4 is strictly dependent on its active-site selenocysteine residue (168), implying that dietary selenium intake directly regulates GPX4 protein expression and functional activity, thereby determining cellular susceptibility to ferroptosis. Within the antioxidant defense system, the fat-soluble vitamin E directly neutralizes lipid radicals within membrane phospholipids, effectively halting the chain reaction of lipid peroxidation. Consequently, when GPX4 function is compromised, vitamin E provides crucial compensatory protection. Research indicates that combined supplementation with selenium and vitamin E synergistically enhances the antioxidant defense capacity of colonic epithelium, reducing ferroptosis levels induced by dysbiosis and thereby significantly alleviating the pathological progression of experimental colitis (169).

Furthermore, the gut microbiota plays a crucial role in selenium metabolism. Specific bacteria, such as Bacteroides and Lactobacillus species, enhance the host intestinal epithelial utilization of selenium by secreting selenoproteins or selenoreductases that convert inorganic selenium into bioavailable organic forms, like selenomethionine (170). In summary, adequate selenium supply effectively inhibits lipid peroxidation and ferroptosis in intestinal epithelial cells by upregulating GPX4 expression, thereby maintaining intestinal barrier integrity. Conversely, selenium deficiency reduces GPX4 activity, increasing cellular susceptibility to ferroptosis and exacerbating intestinal mucosal damage induced by microbial metabolites such as DCA.

#### 5.3.6 Dopamine

Recent studies have demonstrated that certain gut microbes can synthesize dopamine or its precursors through specific metabolic pathways, thereby indirectly regulating host physiological functions, and dopamine can stabilize GPX4 and mitigate ferroptosis-related oxidative stress (171).

In mammals, the central nervous system and enteric chromaffin cells are the primary producers of dopamine. This synthesis process involves sequential catalysis by tyrosine hydroxylase (TH) and aromatic amino acid decarboxylase (AADC), which progressively convert tyrosine into dopamine (172). Furthermore, gut microbiota can produce SCFAs and other metabolites by breaking down dietary fiber, thereby indirectly regulating dopamine synthesis in host intestinal chromaffin cells. Studies further demonstrate that the alleviation of erastin-induced intracellular ferrochrome accumulation and GSH depletion, indicating its potential anti-ferroptotic effects. Clinical observations suggest that decreased intestinal dopamine levels are closely associated with the onset and progression of IBD (173); however, the precise mechanisms underlying this association are not yet fully understood. Notably, dopamine exhibits tissue-specific regulation of iron metabolism and ferroptosis. For instance, in the central nervous system, dopamine may prioritize protecting dopaminergic neurons from ferroptosis, whereas its mechanisms in peripheral tissues, such as the gut, may differ (8). Moreover, dopamine’s biological effects demonstrate dose-dependent characteristics: at low doses, it primarily exerts antioxidant functions, whereas high doses may induce auto-oxidation to generate quinone compounds, which promote ROS production and paradoxically increase the risks of oxidative stress and ferroptosis (174).

#### 5.3.7 Tryptophan

**Figure 2:**
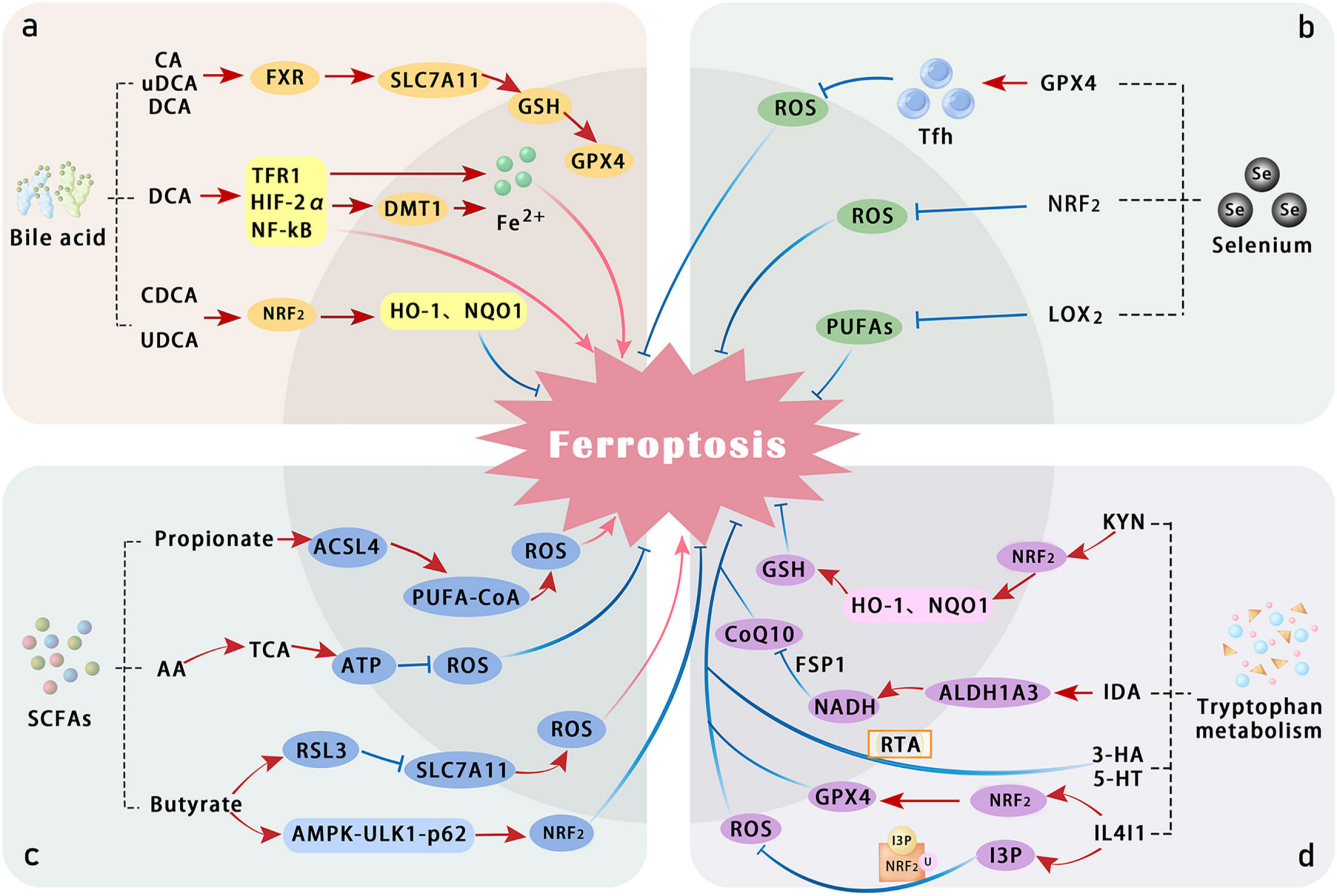
Summary of the mechanisms by which bile acid, selenium, SCFAs, and tryptophan metabolism regulate ferroptosis. **(a)** Several bile acids—such as cholic acid (CA), ursodeoxycholic acid (UDCA), deoxycholic acid (DCA), and chenodeoxycholic acid (CDCA)—activate the Farnesoid X receptor (FXR) and NRF2 pathways, thereby alleviating oxidative stress and attenuating ferroptosis. In contrast, DCA promotes ferroptosis by inducing HIF-2α-mediated labile iron accumulation. **(b)** Selenium counteracts lipid peroxidation directly through GPX4 and may also suppress LOX activity, thereby reducing peroxidation of PUFAs. **(c)** SCFAs, including propionic acid, butyric acid, and acetic acid, modulate cellular antioxidant responses and influence the pathogenesis of colitis. **(d)** Tryptophan and its metabolites suppress ferroptosis through multiple direct and indirect mechanisms involving various metabolic pathways. ALDH1A3, aldehyde dehydrogenase 1 family member A3; ferroptosis suppressor protein 1; IDA, trans-3-indoleacrylic acid; I3P, inositol triphosphate; IL4I1, interleukin-4-induced 1; MT, melatonin; TCA, tricarboxylic acid cycle; Tfh, T follicular helper cells; 5HT, serotonin.

As an essential amino acid, tryptophan plays a pivotal role in metabolic pathways involved in various physiological and pathological processes. The gut microbiota metabolizes tryptophan to produce indole compounds, which inhibit ferroptosis by activating the AhR or exerting direct antioxidant effects. Tryptophan’s regulation of ferroptosis involves mechanisms such as the kynurenine pathway (KP) (**Figure 2**), where host cells generate multiple tryptophan metabolites, including L-kynurenine (L-KYN), kynurenine (KYN), 3-hydroxykynurenine (3-HK), 3-hydroxyanthranilic acid (3-HA), and KYN. These metabolites activate the AhR signaling pathway, conferring ferroptosis resistance to cancer cells and inducing T-cell dysfunction (175). Additionally, gut microbiota regulate tryptophan metabolism to produce indole derivatives and modulate the host’s KP pathway, playing a crucial role in immune homeostasis, neural signaling, and energy metabolism balance. Imbalances in this system have been identified as a daily pathological basis for various diseases (176).

In summary, Metabolomics studies have consistently demonstrated that the intestinal metabolite profiles of IBD patients differ significantly from those of healthy controls, including alterations in short-chain fatty acids (SCFAs), bile acids, and tryptophan-derived metabolites, which are repeatedly observed across independent cohorts (177, 178). Additionally, several studies indicate that although most metabolic changes show common trends in both UC and CD, distinct patterns can also be identified: for example, alterations in the bile acid pool, such as differential shifts in primary versus secondary bile acids. The enrichment of primary bile acids was more obvious in CD. And UC exhibited higher levels of protein fermentation-related metabolites, suggesting these profiles may reflect underlying disease-specific mechanisms (179). While current metabolomic evidence remains insufficient to fully delineate all disease stages or IBD subtypes solely on metabolic profiles, it clearly indicates that individual metabolites exhibit unique association characteristics that may emerge as a critical analytical approach for differentiating disease types and probing underlying mechanisms.

### 5.4 Regulation of gut microbial by ferroptosis

Ferroptosis reshapes the gut microbiota composition by altering the intestinal microenvironment. This may subsequently induce alterations in local gut microbial structure and metabolic products, thereby establishing and exacerbating a vicious cycle of ferroptosis. Relevant mechanisms involve iron metabolism disruption, oxidative stress, intestinal barrier damage, and immune microenvironment imbalance. Under pathological conditions, ferroptosis causes intestinal epithelial damage, characterized by crypt disruption, a reduction in goblet cells, and degradation of tight junction proteins. This compromised barrier function facilitates bacterial translocation while recruiting and activating pro-inflammatory immune cells, such as neutrophils and Th17 cells, thereby intensifying the inflammatory process (180).

Cells undergoing ferroptosis release substantial amounts of labile iron. A high-iron environment promotes the growth of certain pathogenic bacteria while suppressing the growth of beneficial ones. Gu et al. (181) established a high-iron diet mouse model, demonstrating that iron overload significantly increases iron levels in serum, colonic tissue, and feces, and successfully induces colitis phenotypes and ferroptosis. Non-targeted fecal metabolomics analysis revealed significant metabolic differences between iron-deficient, normal, and iron-overloaded groups. Both iron deficiency and iron overload-induced metabolic disorders are closely associated with the genus Dubosiella, with specific genera, such as Akkermansia and Alistipes, showing significant correlations with colitis severity. Iron overload mediates colitis development in mice by simultaneously activating intestinal epithelial cell ferroptosis and disrupting gut microbiota homeostasis. Concurrently, microbial dysbiosis reduces the production of beneficial metabolites such as SCFAs, further compromising intestinal epithelial energy supply and anti-inflammatory defenses.

## 6 Probiotics

**Figure 3:**
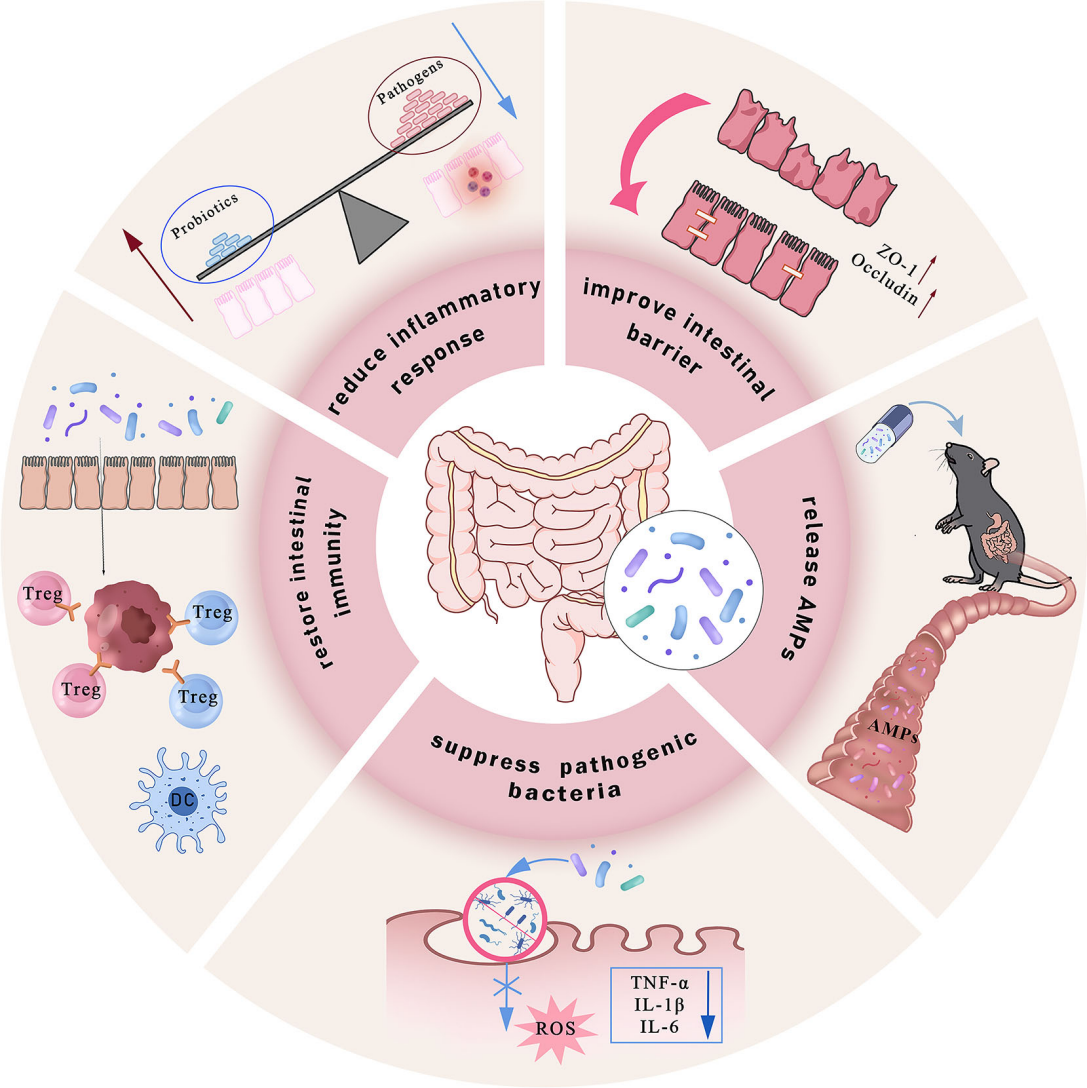
Commensal gut probiotics alleviate IBD through multifaceted mechanisms. Probiotics ameliorate colonic inflammation via coordinated actions, including enhancing intestinal mucosal barrier integrity by upregulating tight junction proteins; directly secreting antimicrobial peptides (AMPs) to neutralize pathogenic bacteria and inhibit their invasion into intestinal epithelial cells; reducing intracellular ROS generation and the secretion of pro-inflammatory cytokines such as TNF-α, IL-1β, and IL-6; competing with pathobionts to restore microbial homeostasis, and modulating intestinal immune responses by promoting regulatory Treg and DCs, while simultaneously suppressing Th17 cell activity. Collectively, these mechanisms reshape the gut microbiota composition and alleviate intestinal inflammation.

Disrupted gut microbiota induces ferroptosis within the gastrointestinal tract. In contrast, probiotic intervention antagonizes ferroptosis through mechanisms such as reducing inflammation, repairing intestinal barrier, releasing antimicrobial peptides (cAMPs), regulating iron overload, and inhibiting lipid peroxidation (182, 183), thereby ameliorating the pathological phenotype of IBD (**Figure 3**). Research indicates that probiotics enhance antibody production through pathways including Toll-like receptor (TLR) activation and helper T cell (Th cell) responses, thereby modulating intestinal mucosal immune system function and participating in the regulation of IBD-associated ferroptosis.

Regarding iron homeostasis regulation, increased levels of Lactobacillus and Bifidobacterium species are correlated with reduced inflammation (184). Both genera can competitively bind free iron in the gut to lower local iron ion concentrations, while simultaneously lowering intestinal pH through the formation of essential amino acids or SCFAs, thereby diminishing iron-dependent lipid peroxidation reactions (185). Furthermore, the probiotic metabolite butyric acid can downregulate intestinal epithelial DMT1 expression and inhibit iron ion influx. Notably, the combined intervention of Clostridium butyricum (C. butyricum) or its metabolite butyrate with ferroptosis-inducing agent RSL3 significantly suppresses pancreatic ductal adenocarcinoma (PDAC) progression. Clinical data also demonstrate a positive correlation between C. butyricum colonization in tumor tissues and a favorable prognosis and low invasiveness in patients with PDAC. This mechanism may involve C. butyricum and its metabolites inducing superoxide stress and intracellular lipid accumulation, thereby enhancing tumor cell susceptibility to ferroptosis (186).

Probiotic intervention significantly enhances the abundance of beneficial bacteria and the α-diversity of the gut microbiota, a key biomarker for clinical remission in inflammatory bowel disease. A. muciniphila (pAKK), a mucosal-associated gut symbiotic bacterium, demonstrates the ability to address multiple metabolic disorders (Zhang et al., 2019). pAKK shows remarkable potential in combating Salmonella infections by upregulating intestinal barrier genes and secreting antimicrobial peptides (187). The outer membrane protein Amuc_1100 (Amuc) is a key bioactive component of Akkermansia muciniphila, contributing to its ability to regulate obesity and maintain gut homeostasis (188). In various disease models, A. muciniphila, also known as Amuc, has been observed to improve intestinal health. Colorectal cancer (189), immune-mediated IBD (190, 191), irritable bowel syndrome (IBS) caused by increased intestinal permeability (192), and intestinal damage and inflammation due to excessive irradiation (IR) during abdominal radiotherapy (193). Research indicates that genetically engineered Lactobacillus gasseri strains exhibiting high expression of specific genes demonstrate more pronounced anti-inflammatory effects in DSS-induced IBD mouse models compared to wild-type strains, suggesting that modifying antioxidant enzymes in probiotics may enhance their anti-inflammatory activity (194).

Dietary factors play a role in regulating IBD progression, with mechanisms likely involving the regulation of gut microbiota by dietary fiber and its impact on intestinal mucosal barrier function. HFD is recognized as a significant risk factor for UC development and progression (195). A prebiotic-rich diet selectively promotes the growth of beneficial gut bacteria, and their metabolic products can be regulated, significantly improving the intestinal microenvironment in IBD. Notably, studies have linked widely used food additives in modern Western diets to the promotion of intestinal inflammation. Polysorbate, a common food emulsifier, has recently been found to enhance the invasiveness of pathogens, facilitating their adhesion to M cells and Peyer’s patches, thereby inducing inflammatory responses (196). Emerging evidence also suggests that artificial sweeteners (NNS) and ultra-processed foods can disrupt the gut microbiota, potentially leading to dysbiosis and increased oxidative stress. This dysbiosis may indirectly promote ferroptosis by impairing microbial-derived antioxidant defenses and enhancing oxidative stress, contributing to IBD progression (197).

These findings provide new insights into the relationship between diet and intestinal inflammation. Targeted delivery of probiotics and their metabolites, based on the pathological link between intestinal microbiota metabolites and ferroptosis, may help inhibit ferroptosis, correct microbiota dysregulation, and restore immune homeostasis, offering a promising precision intervention strategy to alleviate IBD inflammation. Integrating precision medicine into IBD treatment, guided by biomarkers such as inflammatory markers and miRNAs, holds significant potential to optimize therapeutic outcomes. By tailoring treatments to individual profiles, biomarker-driven therapies can enhance efficacy, reduce adverse effects, and provide more personalized care for IBD patients.

## 7 Conclusions and perspectives

In recent years, with the increasing exploration of the intestinal microenvironment, there is growing evidence that dysbiosis of the gut microbiota and disorders of iron metabolism are closely associated with the onset and progression of IBD. Of particular note is ferroptosis, a novel form of programmed cell death, which plays a pivotal role in the pathological process of IBD. As a complex disease driven by multifactorial interactions, the pathogenesis of IBD involves multidimensional pathological mechanisms encompassing genetic susceptibility, immune dysregulation, environmental exposures, and gut microbiota dysbiosis. The dynamic interactive network formed between the gut microbiota and ferroptosis exerts a crucial influence throughout the progression of IBD.

As key regulators of intestinal homeostasis, normal microbiota maintain the integrity of the intestinal epithelial barrier and immune tolerance through their metabolic products. However, during dysbiosis, the abnormal proliferation of pathogenic bacteria not only directly triggers the excessive release of pro-inflammatory factors but also exacerbates the host’s iron metabolism imbalance. On one hand, iron carriers secreted by pathogens competitively sequester free iron within the host, intensifying iron starvation in intestinal epithelial cells. On the other hand, disrupted iron homeostasis permits excess free iron to catalyze lipid peroxidation via the Fenton reaction, thereby activating core ferroptosis regulatory pathways. This vicious cycle of microbiota-induced ferroptosis ultimately leads to intestinal barrier disruption, pathogen translocation, and sustained amplification of the inflammatory cascade.

It is noteworthy that iron metabolism, serving as a bridge linking the microbiota to host pathological processes, exhibits bidirectional regulatory properties: the host’s iron reserve status can significantly influence gut microbiota composition, while specific bacterial strains can reshape host iron distribution by modulating the expression of iron absorption-related proteins. Thus, the interplay among these three components collectively regulates intestinal ferroptosis. This review focuses on the dynamic, interactive network between ferroptosis and the gut microbiota, and its influence on disease progression. It posits that the gut microbiota and its metabolites impact ferroptosis in IBD by regulating intestinal barrier function, modulating inflammatory factors, and maintaining immune homeostasis.

Clinical studies have demonstrated that colonization with specific probiotics is crucial for alleviating clinical symptoms in patients with colitis. Fecal microbiota transplantation (FMT) shows great potential in treating refractory colitis, particularly for recurrent or refractory Clostridium difficile infections, where the core mechanism may involve the donor microbiota’s reprogramming of host iron metabolism. With recent advances in gut microbiota research, Clinicians are now applying personalized fecal microbiota transplantation (FMT) therapies that target the gut-brain axis to treat neuroinflammation. Mechanistically address the question of specific receptors and signaling networks regulating ferroptosis through microbial metabolites, technically explain how to utilize organoids, multi-omics integration, and spatial transcriptomics to analyze *in situ* interactions, and identify biomarkers for predicting ferroptosis and microbial intervention efficacy in clinical applications (198). In line with biomarker-driven precision medicine, recent clinical evidence suggests that stratifying patients by predominant inflammatory pathways (e.g., TNF-α) can meaningfully influence responses to biologics. Relevant studies indicate that TNF-α can serve as a biomarker for IBD (198). supporting the feasibility of “treat-smart” personalized therapy rather than non-specific escalation. Existing preclinical studies in animal models have shown promising results with ferroptosis inhibitors, iron chelation, and microbiota-based therapies such as probiotics, dietary interventions, and fecal microbiota transplantation (FMT). However, the clinical application of these approaches is still in its early stages, and significant gaps remain in our understanding of their feasibility, risks, and challenges. For example, while ferroptosis inhibitors show potential in preclinical models, their safety and efficacy in humans are not yet established. The risk of unwanted side effects, such as oxidative damage due to over-inhibition of ferroptosis, requires careful consideration. Similarly, iron chelation therapies offer promise in regulating iron homeostasis. For instance, the inappropriate use of certain iron chelators may cause liver and kidney damage (199). In patients with IBD, excessive iron removal can lead to malnutrition or other metabolic disorders. Therefore, it is clinically necessary to find an appropriate “balance point” that alleviates inflammation caused by iron overload without impairing normal iron function or inducing adverse effects.

In summary, elucidating the molecular mechanisms by which the gut microbiota influences IBD through ferroptosis not only offers new insights into the disease’s underlying nature but also establishes a theoretical foundation for developing targeted microbiome-based therapeutic strategies. Targeting the ‘gut microbiota-metabolite-ferroptosis’ axis holds significant potential, emerging as an auspicious novel approach for future IBD treatment. Notably, incorporating ferroptosis and microbiota-related biomarkers into such biomarker panels can further optimize patient screening and monitoring. Linking mechanistic understanding with individualized interventions will provide more precise strategies for the clinical treatment of IBD.
